# Metabolomics Analysis Reveals the Alkali Tolerance Mechanism in *Puccinellia tenuiflora* Plants Inoculated with Arbuscular Mycorrhizal Fungi

**DOI:** 10.3390/microorganisms8030327

**Published:** 2020-02-26

**Authors:** Chunxue Yang, Wenna Zhao, Yingnan Wang, Liang Zhang, Shouchen Huang, Jixiang Lin

**Affiliations:** 1 College of Landscape Architecture, Northeast Forestry University, Harbin 150040, China; yangchunxue121@foxmail.com (C.Y.); zwn9426@foxmail.com (W.Z.); yingnan1027@foxmail.com (Y.W.); zl978103903@foxmail.com (L.Z.); suthenyale@foxmail.com (S.H.); 2 Key Laboratory of Saline-alkali Vegetation Ecology Restoration, Ministry of Education, Northeast Forestry University, Harbin 150040, China

**Keywords:** alkali stress, arbuscular mycorrhizal fungi, LC/TOF-MS, metabolomics, *Puccinellia tenuiflora*

## Abstract

Soil alkalization is a major environmental threat that affects plant distribution and yield in northeastern China. *Puccinellia tenuiflora* is an alkali-tolerant grass species that is used for salt-alkali grassland restoration. However, little is known about the molecular mechanisms by which arbuscular mycorrhizal fungi (AMF) enhance *P. tenuiflora* responses to alkali stress. Here, metabolite profiling in *P. tenuiflora* seedlings with or without arbuscular mycorrhizal fungi (AMF) under alkali stress was conducted using liquid chromatography combined with time-of-flight mass spectrometry (LC/TOF-MS). The results showed that AMF colonization increased seedling biomass under alkali stress. In addition, principal component analysis (PCA) and orthogonal projections to latent structures discriminant analysis (OPLS-DA) demonstrated that non-AM and AM seedlings showed different responses under alkali stress. A heat map analysis showed that the levels of 88 metabolites were significantly changed in non-AM seedlings, but those of only 31 metabolites were significantly changed in AM seedlings. Moreover, the levels of a total of 62 metabolites were significantly changed in *P. tenuiflora* seedlings after AMF inoculation. The results suggested that AMF inoculation significantly increased amino acid, organic acid, flavonoid and sterol contents to improve osmotic adjustment and maintain cell membrane stability under alkali stress. *P. tenuiflora* seedlings after AMF inoculation produced more plant hormones (salicylic acid and abscisic acid) than the non-AM seedlings, probably to enhance the antioxidant system and facilitate ion balance under stress conditions. In conclusion, these findings provide new insights into the metabolic mechanisms of *P. tenuiflora* seedlings with arbuscular mycorrhizal fungi under alkali conditions and clarify the role of AM in the molecular regulation of this species under alkali stress.

## 1. Introduction

Soil salinization and alkalization are some of the most severe ecological and agricultural problems throughout the world and severely limit plant growth, development and yield [1,2]. It has been reported that almost 831 million hectares of land have been affected by salt in the soil [3]. In northeastern China, the most remarkable feature is that the soil consists mainly of alkaline salts (e.g., NaHCO_3_ and Na_2_CO_3_), and alkaline meadows cover more than 70% of the land area [4]. Previous studies have demonstrated that alkaline salt stress and neutral salt (e.g., NaCl and Na_2_SO_4_) stress are actually two entirely different stress types [5,6,7]. Alkaline stress not only has the same inhibitory effects as saline stress but also has the unique influence of high pH, which inhibits ion uptake and disrupts the ionic balance of plant cells [8,9]. However, to the best of our knowledge, the majority of studies have emphasized the toxic effects of salt stress and paid little attention to alkaline stress, and the specific alkali-tolerance mechanisms of halophytes are still not fully understood.

*Puccinellia tenuiflora* is a perennial halophyte species in the Poaceae family. It is widely distributed in northeastern China [10]. This grass has high nutritional value for livestock because of its high crude protein and low crude fiber and ash levels [11]. In addition, this grass also has high tolerance to extremely salt-alkali soil (pH: 9–10) and is considered to be one of the most promising grass species for grassland rehabilitation in northern China. 

Previous reports have revealed some mechanisms by which *P. tenuiflora* seedlings survive under alkaline stress. To cope with stress conditions, this plant has developed various adaptive strategies, such as regulating osmotic adjustment, maintaining ion balance and scavenging reactive oxygen species [12,13]. *P. tenuiflora* can significantly accumulate organic acids, especially citric acid, in both leaves and roots under alkali stress [13]. In addition, some ion salt-alkali-responsive genes encoding antiporters/channels, including *PutAKT1*, *PutCAX1* and *PutNHA1* of this species have been transformed into other plants, such as rice and Arabidopsis, and their biological functions were tested [14,15,16]. Yu et al. (2013) found 43 differentially expressed proteins in *P. tenuiflora* seedlings under alkali stress using proteomic technology, and the results suggested that their alkali response mechanisms include a decline in photosynthesis, the activation of multiple antioxidant mechanisms and an enhanced energy supply [17]. However, the positive role of arbuscular mycorrhizal fungi has been overlooked when analyzing the alkali tolerance mechanisms of *P. tenuiflora*. Alkali tolerance in some halophytes and mycorrhizal symbiosis are closely related in the natural environment.

Arbuscular mycorrhizal fungi, one of the most important groups of soil microorganisms, can colonize roots of more than 80% of terrestrial plants to establish symbiotic associations [18]. They can not only expand the root absorption area and increase the uptake of nutrients (especially phosphorus) for plants, but also improve plant tolerance of environmental stresses (salt-alkali/drought stress, water stress, nutrition stress and temperature stress) by accumulating compatible solutes and maintaining osmotic homeostasis [19,20,21,22,23,24]. In addition, some studies have already demonstrated the molecular mechanisms of arbuscular mycorrhizal fungi (AMF) in enhancing the ability of plants to resist salt stress. For example, Ouziad et al. (2006) reported that the colonization of tomato seedlings by AMF resulted in a drastic increase in the mRNA of all three aquaporin genes assayed under salt stress, and AMF controlled aquaporin expression and thereby presumably regulated water flow in tomato under salt stress [25]. He et al. (2011) also reported that AMF inoculation induced a high level of expression of aquaporin genes in tomato seedlings under salt stress, and AMF also increased the relative water content, water potential and hydraulic conductance [26]. Chen et al. (2018) found that AMF improved the salt tolerance of black locust by increasing K^+^/Na^+^, water status and photosynthetic capacity [27]. The improvement in photosynthesis from AMF was mainly related to the higher expression of three chloroplastic genes (*RprbcL*, *RppsbA*, and *RppsbD*) compared with that in non-AMF plants. However, to the best of our knowledge, the molecular mechanisms of plant-AMF interactions under alkali stress are still unknown.

Metabolomics is a developing and promising functional genomics tool that describes the metabolic changes and complex molecular interactions that occur in organisms [28,29]. In general, metabolites are the end products of cellular regulatory processes, and the amount of metabolites can be considered the ultimate response of plant to environmental changes [30]. The metabolic balance in plants is constantly disrupted and ultimately changed under stress (e.g., salt-alkali) environments. In addition, gene and protein expression levels are also changed dramatically in the metabolic process of plants under such conditions. The application of metabolomics methods offers a rational way to reveal the implications of these metabolic changes on a broad scale [31]. Currently, metabolomics-based technologies have been broadly employed to clarify the metabolic responses of various plants (both halophytes and glycophytes) to salt stress, such as *Hordeum vulgare* [32], *Oryza sativa* [33], *Suaeda corniculata* [34], *Glycine max* [30], and *Zea mays* [35]. These studies accelerate the understanding of the molecular mechanisms of plant salt tolerance at the metabolic level. However, there is very limited research based on metabolomics that reveals the metabolic reactions of plant-AMF symbionts, especially those of the high-quality halophyte *P. tenuiflora* with AMF, under alkaline stress.

In the present study, the alkali-responsive physiological and metabolomic features in *P. tenuiflora* seedlings with or without AMF were investigated. We aimed to explore whether AMF colonization could enhance the alkali tolerance of this species and to clarify the alkali tolerance mechanism in arbuscular mycorrhizal *P. tenuiflora* plants.

## 2. Materials and Methods

### 2.1. Experimental Design

A controlled pot experiment with a completely randomized design was carried out in the greenhouse of Northeast Forestry University (Harbin, China) in 2017. The controlled environmental conditions were as follows: 55%–65% relative humidity, 14 h light (700 mmol·m^−^^2^·s^−^^1^) daily and an average temperature of 24 °C. The experiment employed two factors: AMF treatment (*Rhizophagus intraradices*, BGC AH01, provided by the Institute of Plant Nutrition and Resources, Beijing Academy of Agriculture and Forestry Sciences, China) and alkali stress (AS) treatment (0, 100, 200 and 300 mM NaHCO_3_). Each treatment was conducted in three replicates, and one pot with eight seedlings was considered to represent one replicate.

### 2.2. Plant Culture and Stress Treatments

Seeds of *P. tenuiflora* were collected from the Songnen grassland in Heilongjiang Province, China (E 125°42′, N 46°16′). The seeds were surface-sterilized with 10% sodium hypochlorite for 10 min, rinsed three times with distilled water and then sown in plastic pots that were filled with 2 kg of an autoclaved soil mixture (soil: sand 3:1, *v*/*v*). Before this step, the substrate was autoclaved at 121 °C and 240 kPa pressure for 2 h, to ensure that all possible mycorrhizal propagules and other microorganisms had been destroyed. Each pot was inoculated with either 25 g of the inoculant for the inoculation treatment or 25 g of sterilized inoculant for the non-inoculation treatment. We also added 30 mL of filtered inoculant (0.25-μm filter membrane) that was free of mycorrhizal propagules to the -AM treatment to maintain the same microorganism biota. The inoculants were added 2 cm below the seeds. The alkali stress treatments were implemented by adding the corresponding solutions as described above 80 days after sowing, and the control seedlings were irrigated with 300 mL of distilled water. The positions of the pots were changed every day to eliminate environmental effects. The seedlings were harvested 10 days after the treatments were implemented.

The harvested seedlings were washed with distilled water three times. The leaves and roots of each plant were separated. The leaves were oven-dried at 105 °C for 15 min and then dried at 65 °C to a constant weight, and the dry weights (DW) were then determined. The roots were measured for mycorrhizal colonization. A fraction of the roots was carefully washed, cut into 1 cm long segments, cleaned with 10% KOH solution and stained with 0.05% trypan blue in lactophenol [36]. AM colonization was quantified according to the formula described by Wu et al. (2015) [37].

### 2.3. Metabolomics Analysis

Metabolite Extraction and Profiling Analysis

Fresh shoot samples (0.1 g, 200 mM NaHCO_3_ treatment) were extracted in prechilled methanol (3 mL) and then centrifuged for 20 min at 4000× *g* after 30 min of sonication. Finally, the supernatant was absorbed through a 0.22 μm filter for liquid chromatography combined with mass spectrometry (LC-MS) analysis. The samples were separated and quantified following the method of Zhang et al. (2016) [38]. In brief, the extracts were separated on a Ultra Performance Liquid Chromatography (UPLC) system (Waters Corporation, Japan). The UPLC column was an ACQUITY UPLC C18 column (2.1 μm, 2.1 mm × 100 mm), which was maintained at 35 °C and eluted with a multistep gradient over the course of 30 min at 0.4 mL/min. The gradient was composed of A (formic acid-water, 0.1/99.9 (*v*/*v*)) and B (formic acid-acetonitrile, 0.1/99.9 (*v*/*v*)) as follows: 0–5 min, isocratic gradient at 5% B; 5–50 min, linear gradient from 5% to 100% B; 50–60 min, isocratic gradient at 100% B. The time-of-flight mass spectrometry (TOF-MS) parameters were set and performed as follows: in positive and negative ionization mode; 60 psi of nebulizer pressure, 720 L/h of drying gas flow, 450 °C of source temperature. The capillary voltages were 5000 V and 4500 V in positive and negative modes, respectively.

### 2.4. Data Processing and Multivariate Data Analysis

The molecular feature extraction (MFE) algorithm for automated peak detection and the chromatographic deconvolution of Mass Hunter software (Agilent Technologies, Santa Clara, CA, USA) were used to acquire and preprocess the data. Peaks with ratios (signal/noise) of <5 were removed. The data were analyzed using SIMCA software (V14, Umetrics AB, Umea, Sweden) for multivariate statistical analysis (principal component analysis (PCA) and orthogonal projections to latent structures discriminant analysis (OPLS-DA)). Subsequently, the KEGG (http://www.genome.jp/kegg/) and NIST (http://www.nist.gov/index.html) were used to construct the metabolic pathway. MetaboAnalyst 4.0 (www.metaboanalyst.ca/) was employed to perform pathway analysis and build heatmap diagrams. The iPath v3.0 web server (https://pathways.embl.de/) was used to build a model of the metabolite profiles, which was performed quantitatively by mapping the KEGG annotated metabolites onto metabolic pathway maps.

### 2.5. Statistical Analysis

The data from three biological replicates were analyzed using the statistical software SPSS 13.0 (SPSS Inc., Chicago, IL, USA) and represented as the mean ± standard error. One-way analysis of variance (ANOVA) was used to test the differences between each treatment group and the control followed by Tukey’s post hoc test. *p* < 0.05 was considered significant. 

## 3. Results

### 3.1. Root Colonization and Seedling Growth

**Figure 1:**
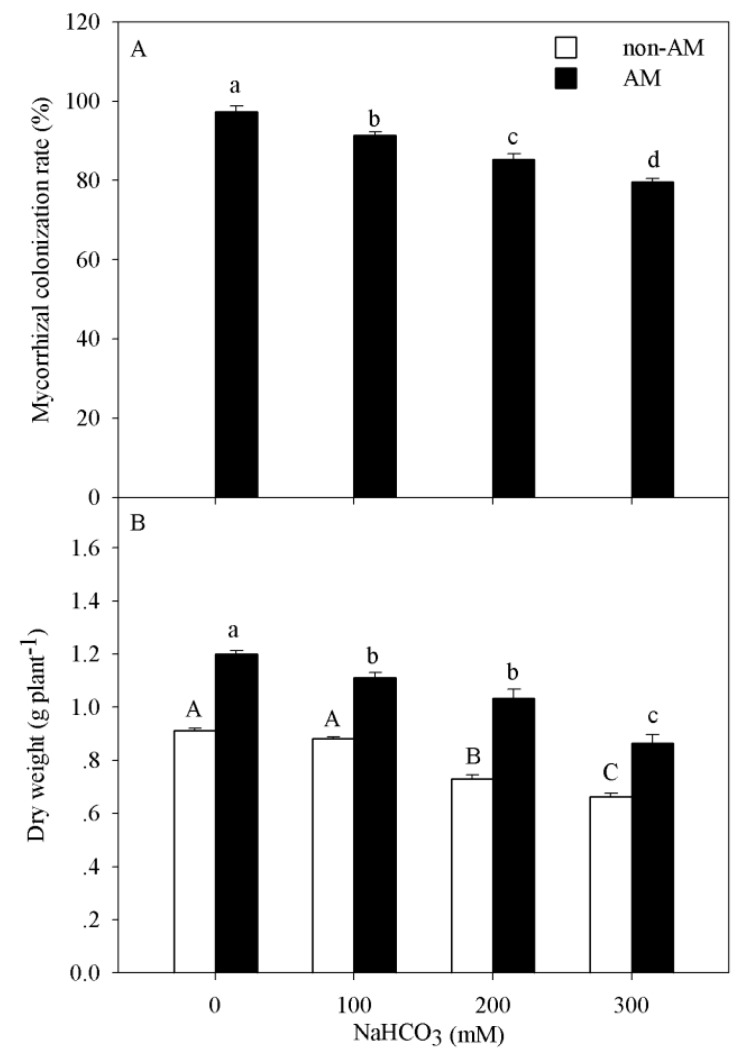
Mycorrhizal colonization rate (**A**) and growth (**B**) of *P. tenuiflora* seedlings under alkali stress. The bars represent the means ± S.E. The differences were considered significant when *p* < 0.05 and indicated by different letters.

The colonization rate significantly decreased with increasing alkali concentration (*p* < 0.05). The maximum colonization rate (97.33%) was obtained in the control treatment (Figure 1A). Two-way ANOVA results showed that dry weight of seedlings was affected by the alkalinity concentration (*p* < 0.001) and the presence of arbuscular mycorrhizal fungi (*p* < 0.001, Table 1). The dry weights of seedlings with AM and seedlings without AM (CK) both decreased significantly with increasing alkali concentration (*p* < 0.05). For example, the dry weight decreased 27.4% and 28.3% at the highest alkalinity (300 mM) compared to that in the control in the non-AM seedlings and AM seedlings, respectively. In addition, the dry weight of AM seedlings was much higher than that of non-AM seedlings, both in the control and under alkali stress (Figure 1B).

### 3.2. Metabolic Profiling

**Figure 2:**
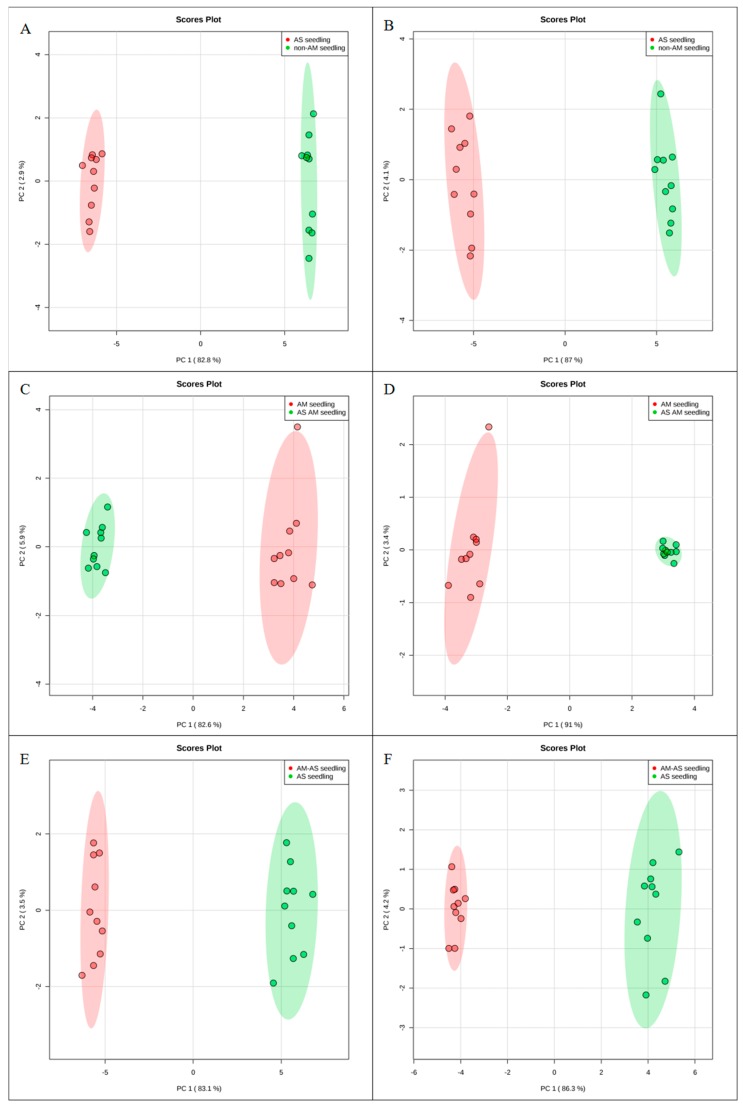
Principal component analysis (PCA) score plots of metabolic profiles in non-AM seedlings in response to alkali stress (**A**,**B**), AM seedlings in response to alkali stress (**C**,**D**) and comparison in non-AM seedlings and AM seedlings under alkali stress (**E**,**F**) in positive (**A**,**C**,**E**) and negative modes (**B**,**D**,**F**). *PC1* and *PC2* are the first two principal components.

**Figure 3:**
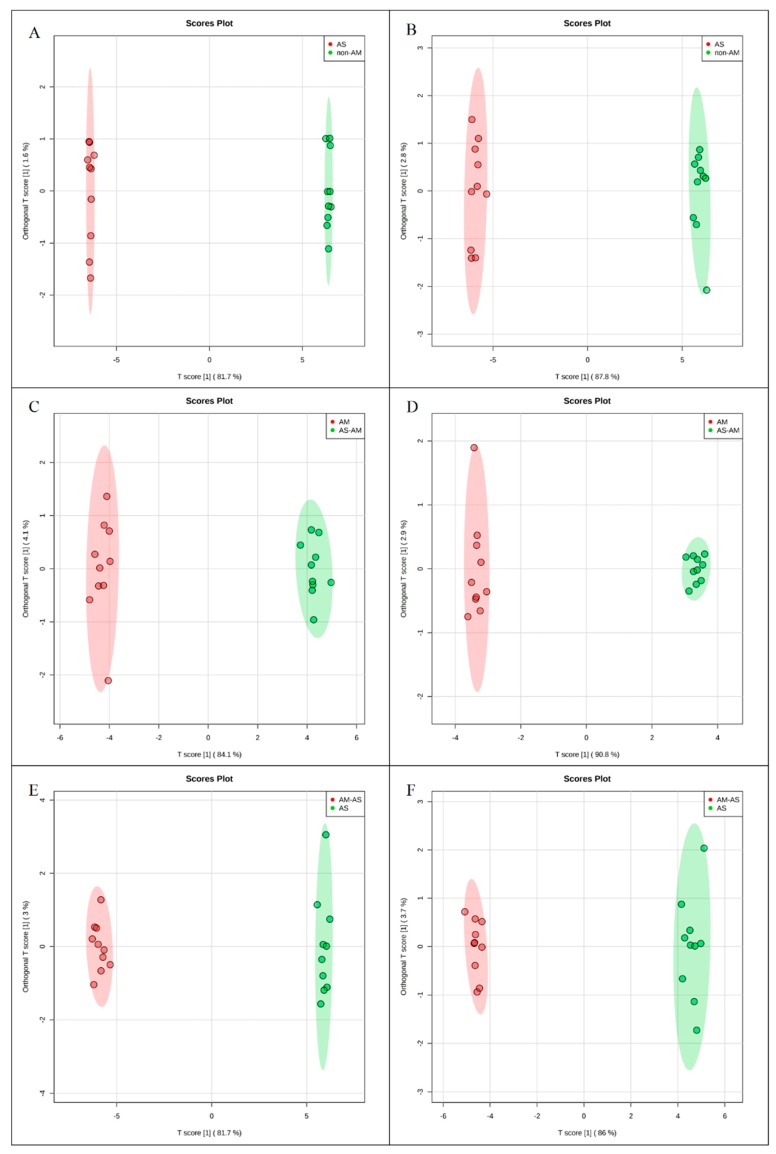
Orthogonal projections to latent structures-discriminate analysis (OPLS-DA) score plots of metabolic profiles in non-AM seedlings in response to alkali stress (**A**,**B**), AM seedlings in response to alkali stress (**C**,**D**) and comparison in non-AM seedlings and AM seedlings under alkali stress (**E**,**F**) in positive (**A**,**C**,**E**) and negative modes (**B**,**D**,**F**).

To investigate the molecular mechanisms of *P. tenuiflora*-AMF symbionts, 80-day-old seedlings treated with 200 mM NaHCO_3_ for 10 days were analyzed by metabolomics with the liquid chromatography-mass spectrometry technique. The ESI-TOF-MS data were analyzed by principal component analysis (PCA) and orthogonal projections to latent structures-discriminant analysis (OPLS-DA). A PCA score plot showed the distributions of the origin data, and the samples were separated into AS and CK (Figure 2A,B), AM-AS and AM (Figure 2C,D), and AM-AS and AS (Figure 2E,F) groups under the positive (Figure 2A,C,E) and negative (Figure 2B,D,F) modes, respectively. In addition, we performed pairwise comparisons of the data obtained by OPLS-DA, whose score plot exhibited an obvious distinction among the AS and CK (Figure 3A,B), AM-AS and AM (Figure 3C,D), and AM-AS and AS (Figure 3E,F) groups under positive mode (Figure 3A,C,E) and negative mode (Figure 3B,D,F), respectively. Three parameters, R^2^X, R^2^Y and Q^2^Y, were used to evaluate the models for similarity and predictability (Figure S1). The R2X, R2Y, and Q2Y in the AS and CK (Figure S1A,B), AM-AS and AM (Figure S1C,D), and AM-AS and AS (Figure S1E,F) groups indicated that the OPLS-DA models were of good enough quality to have satisfactory predictive power.

**Figure 4:**
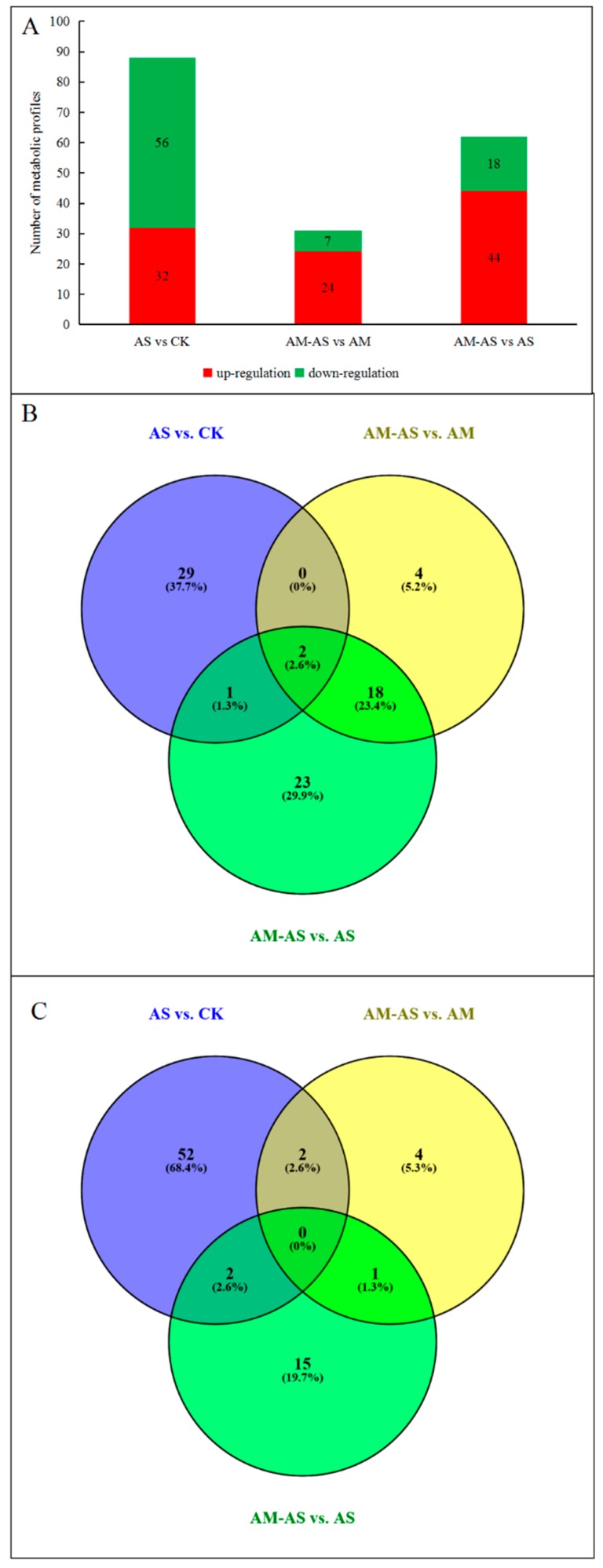
Identification and statistics of metabolic changes under alkali stress. (**A**) Number of up-or down-accumulated metabolic profiles between AM seedlings and non-AM seedlings of *P. tenuiflora* under alkali stress. (**B**) Venn diagram analysis of up-accumulated metabolic profiles in non-AM seedlings and AM seedlings under alkali stress. (**C**) Venn diagram analysis of down-accumulated metabolic profiles in non-AM seedlings and AM seedlings under alkali stress. CK: control treatment, AS: alkali stress without arbuscular mycorrhizal, AM: arbuscular mycorrhizal treatment, AM-AS: alkali stress with arbuscular mycorrhizal.

The metabolic profiles identified for the AM and non-AM *P. tenuiflora* seedlings were quantitatively analyzed. In the metabolic profiles, the most relevant metabolites in response to alkali stress or inoculation with AMF were considered to be those whose variable importance in the projection (VIP) values were greater than 1 and *p*-values were lower than 0.05. In the present study, 32 metabolites were identified as having increased concentrations and 56 metabolites had decreased concentrations in the AS and CK groups. Twenty-four metabolites were identified as having increased concentrations and seven metabolites had decreased concentrations in the AM-AS and AM groups. In addition, 44 metabolites were identified as having increased concentrations and 18 metabolites had decreased concentrations in the AM-AS and AS groups (Figure 4A). The results reflected that more metabolites displayed changes in their levels in the non-AM seedlings. To identify the common and specific types of metabolites between AM seedlings and non-AM seedlings, a Venn diagram was generated (Figure 4B,C). The diagram showed that two common metabolites (C01234 and C00956) reflected a similar variation trend in non-AM seedlings and AM seedlings of this species under alkali stress (Figure 4B,C). In addition, the metabolic model was used to determine the completeness of metabolic pathways. The models reflected that the non-AM seedlings and the AM seedlings used different metabolic pathways in response to alkalinity stress (Figures S4 and S5).

### 3.3. Metabolic Changes in the Non-AM Seedlings of P. tenuiflora under Alkali Stress

A significant variance (VIP > 1, *p* < 0.05) in the metabolic profiles was observed in the non-AM seedlings under alkali stress, and a total of 88 metabolites with significant differences from the control were identified (Figure 4A), including amino acids and amines, carbohydrates and polyols, inorganic and organic acids, fatty acids, flavonoids, steroids and sterol, nucleic acids, phytohormones and others (Table S1). The metabolites that showed more than 15-fold changes in concentration in non-AM seedlings under alkali stress included hydromorphone (21.38-fold) and ethylmorphine (20.02-fold). Those with changes in the 5–15-fold range included isomaltose (6.21-fold), pyridoxine (−6.66-fold), galactinol (7.77-fold), DL-2-aminoadipic acid (−9.09-fold), formylanthranilic acid (−9.09-fold), quercetin 3′-methyl ether (13.79-fold), apigenin 7-*O*-neohesperidoside (−12.5-fold) and allantoin (−7.14-fold). The rest of the metabolites showed concentration changes in the 1–5-fold range (Table S1). The heat map analysis showed that the metabolites were significantly decreased in non-AM seedlings under alkali stress (Figure S2).

### 3.4. Metabolic Changes in AM Seedling of P. tenuiflora under Alkali Stress

In AM seedlings, 31 metabolites had significantly changed concentrations under alkali stress (Figure 4A). The metabolites that showed concentration changes in the 5- to 15-fold range included quinate (8.02-fold), apigenin 7-*O*-neohesperidoside (6.88-fold) and allantoin (6.22-fold). Noticeably, the concentrations of many amino acids and amines, inorganic and organic acids and flavonoids were significantly increased in the 1–5-fold range in AM seedlings under alkali stress (Table S2). The heat map showed that the levels of most metabolites in AM seedlings increased in response to alkali stress (Figure S3).

### 3.5. Comparison of Metabolic Patterns in Non-AM and AM Seedlings under Alkali Stress

**Figure 5:**
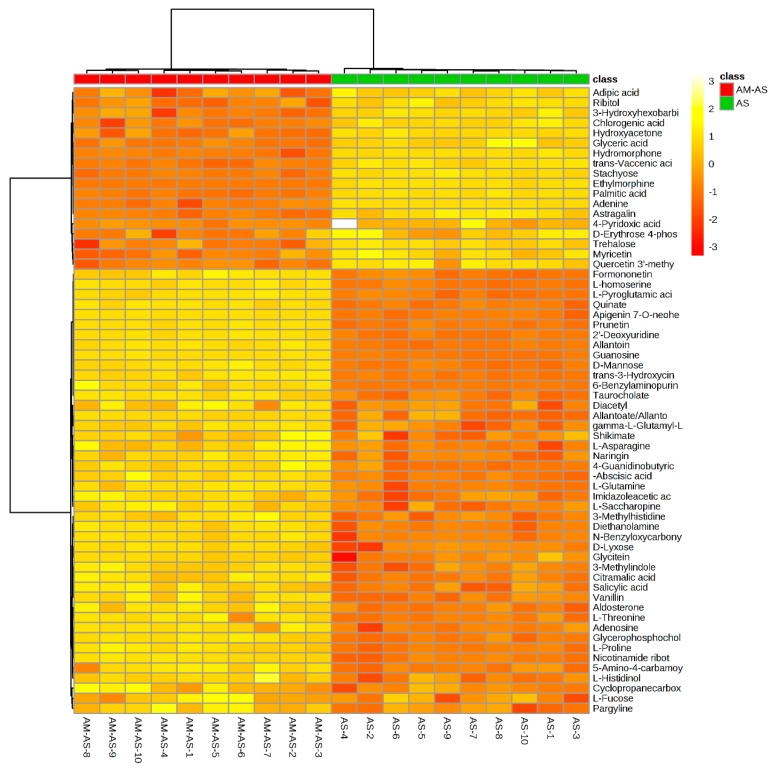
Heat map analysis combined with hierarchical cluster analysis in comparison between alkali stress with AM and only alkali stress treated seedlings of *P. tenuiflora*. AS: alkali stress without arbuscular mycorrhizal, AM-AS: alkali stress with arbuscular mycorrhizal.

To further understand the mechanisms of *P. tenuiflora* seedlings with or without AM under alkali stress, the metabolites under alkali stress in the AM treatment (AS-AM) and the alkali stress treatment (AS) were compared. The concentrations of 62 metabolites were found to be significantly different between AM and non-AM plants under alkali stress (Figure 4A). Only one metabolite with a greater than 15-fold change in concentration was found between non-AM seedlings and AM seedlings under alkali stress, quercetin 3′-methyl ether (−16.67-fold). The metabolites that showed differences in the 5–15-fold range included 3-methylhistidine (6.78-fold), apigenin 7-*O*-neohesperidoside (9.26-fold), allantoin (8.15-fold), hydromorphone (−14.28-fold), ethylmorphine (−14.29-fold) and 6-benzylaminopurine (5.64-fold) (Table 2). The heat maps revealed that most metabolites, including amino acids and amines, nucleic acids and phytohormones, were increased in *P. tenuiflora* seedlings under alkali stress after mycorrhizal inoculation (Figure 5). Notably, the metabolites involved in osmotic adjustment (such as proline) and reactive oxygen species scavenging (l-glutamine and gamma-l-glutamyl-l-glutamic acid) were significantly increased in *P. tenuiflora* seedlings under alkali stress after inoculation (Table 2 and Figure 5).

## 4. Discussion

### 4.1. Arbuscular Mycorrhizal Fungi Colonization and Seedling Growth

Previous studies have suggested that alkali stress could inhibit spore germination and hyphal growth and lead to a reduction in colonization [39]. In the present study, we also found that colonization decreased with increasing alkalinity (Figure 1A). However, the colonization rate remained high at the 300 mM NaHCO_3_ concentration, indicating that arbuscular mycorrhizal fungi can establish a good symbiotic relationship with *P. tenuiflora* under alkali stress.

Seedling establishment is one of the most sensitive and important stages of plant development in alkali-degraded soil [40]. In the present study, the dry weight of *P. tenuiflora* seedlings with or without AM decreased. However, *P. tenuiflora* seedlings inoculated with *R. intraradices* had higher dry weights than non-AM seedlings under all conditions, indicating that AM fungus improved the alkali tolerance of *P. tenuiflora* seedlings (Figure 1B). Similar results were also found in *Zea mays* [41], *Lactuca sativa* [42] and *Leymus chinensis* [4].

### 4.2. Metabolic Differences: The Responses of P. tenuiflora Seedlings with or without Arbuscular Mycorrhizal Fungi to Alkali Stress (AM-AS vs. AM, AS vs. CK)

Previous studies have reported that the primary and secondary metabolism in plants is affected by arbuscular mycorrhizal fungi [43,44]. In the present study, the metabolites and their associated metabolic pathways in AM-seedlings and non-AM seedlings were examined based on metabolomics under alkalinity stress. The results showed that 88 metabolites were significantly changed in non-AM seedlings, but only 31 metabolites in AM seedlings (Figure 4A). In addition, the Venn analysis of changed metabolites in AM-seedlings and non-AM seedlings showed that only two common metabolites (C01234 and C00956) reflected a similar variation trend at alkali stress (Figure 4C). The results suggested that *P. tenuiflora* seedlings with and without AM have different mechanisms in response to alkali stress (Figures S4 and S5).

In detail, the quantitative analyses revealed that a large number of amino acids and amines decreased in the *P. tenuiflora* seedlings without AM (Table S1). Less et al. (2008) reported that free amino acids were not only the basic unit of protein synthesis but also had a significant effect on stress resistance in plants [45]. As a result, the decreased amino acid and amine contents in non-AM seedlings indicated that alkali stress inhibited protein synthesis and then disturbed the normal physiological metabolism of this species. However, the amino acid and amine contents were increased in AM seedlings of *P. tenuiflora* under alkali stress, indicating that mycorrhizal colonization improved the resistance of these seedlings to alkali stress (Table S2).

Previous studies reported that small molecular carbohydrates and polyols accumulated under salt stress and acted as compatible solutes to improve salt tolerance in plants [46,47]. Consistent with previous studies, most carbohydrates and polyols (e.g., sucrose, D-lactose and stachyose) in non-AM seedlings accumulated under alkali stress. However, only three metabolites involved in carbohydrate and polyol metabolism changed in AM seedlings. This result indicates that different physiological responses occur in AM seedlings and non-AM seedlings of *P. tenuiflora* and that improving carbohydrate and polyol metabolism is an important way to alleviate alkali stress in non-AM seedlings of this species. 

Some studies have previously reported that organic acid metabolism could help many plants, such as *Chloris virgata* [5], *Suaeda glauca* [48] and *Leymus chinensis* [49], adapt to alkali stress. Organic acid contents increase to balance the osmotic pressure and stabilize the intracellular pH under alkali stress. In contrast to the previous results, our study showed that most organic acids in non-AM seedlings of *P. tenuiflora* significantly decreased (*p* < 0.05), and only three organic acids (citrate, fumaric acid and malic acid) involved in the TCA cycle accumulated under alkali stress. However, we could not show that the organic acids involved in the TCA cycle were changed in the AM seedlings of this species. The TCA cycle is an important energy-producing process in plants, and plays an important role in resisting stress conditions [50]. The results revealed that the TCA cycle was only significantly enhanced in non-AM seedlings under alkali stress, indicating that the enhancement of energy capacity in non-AM seedlings could compensate for the energy lost due to stress. This compensation mechanism would help seedlings cope with alkali stress.

Our results showed that the fatty acid contents increased in non-AM seedlings but remained unchanged in AM seedlings of *P. tenuiflora* under alkali stress. The saturated fatty acid was stearic acid, and the unsaturated fatty acids were arachidonic acid, oleic acid and alpha-linolenic acid. In general, β-oxidation is the primary method of fatty acid decomposition, which provides a large amount of energy for life activities and plays an important role in plant stress responses. Therefore, the accumulation of fatty acids may prompt β-oxidation in non-AM seedlings and generate large amounts of energy, which would reduce the damage caused by alkali stress. However, the present study also showed that β-oxidation is not the main metabolic pathway for AM seedlings under alkali stress. Previous studies have reported that sterol plays an important role in maintaining membrane permeability and fluidity, which are both affected by salt stress [51]. In this study, the sterol content decreased in non-AM seedlings but was unchanged in AM seedlings under alkali stress. The results indicated that alkali stress induced oxidative damage by the generation of reactive oxygen species (ROS) in non-AM seedlings, which destroyed membrane lipids and ultimately led to a high level of electrolyte leakage in non-AM seedlings.

### 4.3. Effects of Arbuscular Mycorrhizal Fungi on the Metabolic Profiles of P. tenuiflora Seedlings under Alkali Stress (AM-AS vs. AS)

Metabolites, which are the end products of all cellular processes and the basis for phenotype expression, can affect gene transcription and protein expression in turn [52]. Furthermore, the metabolite responses of plants after inoculation with AMF under alkali stress included both plant-related and AM-related metabolites. Plant-related metabolites are highly abundant “background” metabolites that are not involved with AMF but show differential abundance under alkali stress. Hence, we investigated the differences of metabolites between *P. tenuiflora* seedlings with arbuscular mycorrhizal fungi under alkali stress and *P. tenuiflora* seedlings under alkali, to explore the mechanisms by which AMF enhance the alkali tolerance of *P. tenuiflora* seedlings. This is an effective way to remove the influence of these “background” metabolites and is very helpful for identifying the desired AM-related metabolites. Sixty-two metabolites changed significantly between alkali stress with AM treatment and alkali stress treatment alone (AM-AS vs. AS). These metabolites were derived from nine metabolite pathways, including amino acids and amines, carbohydrates and polyols, organic acids, fatty acids, flavonoids, steroids, nucleic acids, phytohormones and others (Figure 4A and Figure S6 and Table 2).

The increase in amino acid metabolism and the accumulation of small molecule amino acids enhance the salt tolerance of plants by improving osmotic adjustment and maintaining cell membrane stability [53]. The present study showed that amino acid metabolism significantly increased after inoculation with AMF under alkali stress, leading to a significant accumulation of glutamine, proline, asparagine, threonine, homoserine, saccharopine, 3-methylhistidine, pyroglutamic acid and gamma-l-glutamyl-l-glutamic acid. Tavakoli et al. (2018) reported that proline accumulation could protect enzyme activity, reduce soluble protein precipitation, decrease intracellular osmotic potential and balance protoplast osmotic pressure [54]. In addition, Jiao et al. (2018) reported that the GS/GOGAT cycle played an important role in proline synthesis under salt stress [55]. In the present study, we found that the intermediate metabolites of the GS/GOGAT cycle (l-asparagine and gamma-l-glutamyl-l-glutamic acid) accumulated and ultimately led to an enhancement in proline content after inoculation under alkali stress. Moreover, increases in the metabolism of carbohydrates, polyols and amino acids, which are the main osmotic adjustment substances of plants in response to salt stress, have been reported in several plants, such as rice [56], wheat [57] and poplar [58]. In this study, carbohydrate and polyols (mannose, lyxose, histidinol and fucose) accumulated after inoculation with AMF under alkali stress. These results suggested that mycorrhizal status increased the carbohydrate and polyol contents in *P. tenuiflora* seedlings to enhance their osmotic adjustment with amino acids, which helps seedlings stabilize their membrane structures under alkali stress.

Organic acids, as small-molecule osmotic adjustment substances, play an important role in the physiological process of salt resistance, especially under alkali stress [59]. Our results showed that the levels of some organic acids increased significantly after AMF inoculation under alkali stress. These organic acids included shikimic acid, allantoic acid, 4-guanidinobutyric acid, cyclopropanecarboxylic acid, imidazoleacetic acid trans-3-hydroxycinnamic acid and citramalic acid. Shikimic acid is a plant metabolite that participates in the synthesis of membrane phospholipids [60]. Phosphatidylethanolamines and lignin are used in the synthesis of plant cell membranes and cell walls [61]. The accumulation of shikimic acid in this study indicated that AMF could promote cell membrane and wall growth and relieve the growth inhibition of *P. tenuiflora* seedlings under alkali stress. Purine catabolism is regarded as a housekeeping function that remobilizes nitrogen for plant growth and development [62]. In addition, plants induce and activate enzymes by the purine degradation pathway in response to abiotic and biotic stresses [63]. This activation often results in an increasing level of intermediary metabolites, particularly allantoic acid, under stress conditions [64]. In the present study, the allantoic acid content increased after AMF inoculation, indicating that AMF improved purine catabolism and then enhanced nitrogen utilization, which might lead to the accumulation of N-containing compounds (such as amino acids and organic acids) in the cytoplasm and ultimately an increase in alkali tolerance in *P. tenuiflora* seedlings.

In the present study, we found that the fatty acid contents decreased after inoculation but remained unchanged in AM seedlings under alkali stress. The results indicated that removing the plant-related “background” metabolites was effective and could distinguish AMF-related proteins in order to explore the mechanisms by which AMF enhanced the alkali tolerance of *P. tenuiflora* seedlings.

Flavonoids can enhance tolerance to abiotic and biotic stresses in plants due to their ability to remove dangerous stress-response substances from the cell, including free radicals, singlet oxygen molecules and peroxides [65]. The results in the present study showed that most flavonoids were significantly increased after inoculation with AMF under alkali stress. These flavonoids included naringin, prunetin, apigenin 7-*O*-neohesperidoside, glycitein and formononetin. These results suggest that AM fungi improved the accumulation of flavonoids in *P. tenuiflora* seedlings under alkali stress, which might increase their antioxidant capacity and result in an increase in alkali tolerance in *P. tenuiflora* seedlings [66].

The synthesis of sterols is considered an important method by which plants develop and survive under various types of stress conditions [67]. Sterols in plants form a basic structural unit of membranes that allows the membranes to be permeable to fluids and essential components [68]. In the present study, the aldosterone and taurocholate contents in seedlings increased significantly after inoculation with AMF under alkali stress. The previous results of our study demonstrated that alkali stress induced oxidative damage by generating reactive oxygen species (ROS) and resulted in an increase in electrolyte leakage and membrane damage in the non-AM seedlings of *P. tenuiflora*. However, inoculation with AMF increased the sterol content in seedlings under alkali stress, indicating that AMF enhanced membrane permeability and fluidity and ultimately enhanced alkali tolerance in *P. tenuiflora* seedlings.

Plant hormones are considered essential endogenous molecules that are involved in regulating plant development and stress tolerance [69]. Salicylic acid is a plant endogenous signal molecule that can enhance the antioxidant system, remove excess reactive oxygen species and reduce the level of membrane lipid peroxidation in plant cells. Salicylic acid can also increase metabolic activity and alleviate the inhibition of salt stress on plants [70,71]. Abscisic acid is also known as a plant stress hormone that is involved in regulating several plant stress responses. It is one of the main components that ensure stomatal closure in response to unfavorable environments. In addition, abscisic acid can also protect membrane integrity and facilitate ion regulation, uptake and transport in plants under salt stress. In this study, the contents of salicylic acid and abscisic acid were both increased after inoculation with AMF under alkali stress. Similar results were also found in *Cucumis *sativus** and *Lycium barbarum* [72,73]. The results showed that AMF significantly changed the levels of various plant hormones, which was an effective way to enhance alkali tolerance in *P. tenuiflora* seedlings.

## 5. Conclusions

In brief, this study presents the first investigation into the effects of AMF colonization on the metabolic profile of *P. tennuiflora* under alkali stress. The results clearly show that AMF inoculation significantly increased amino acid, organic acid, flavonoid and sterol contents in order to enhance osmotic adjustment and maintain cell membrane stability under alkali stress. *P. tenuiflora* seedlings after AMF inoculation produced more plant hormones (salicylic acid and abscisic acid) to enhance antioxidant systems and facilitate ion balance under stress conditions. These findings provide new insights into the metabolic mechanisms of alkali stress in *P. tenuiflora* seedlings with AMF and clarify the role of AMF in the molecular regulation of this species under alkalinity stress.

## Figures and Tables

**Table 1:** ANOVA effects of alkalinity concentration (A), arbuscular mycorrhizal fungi (AMF) and their interactions on the growth of *Puccinellia tenuiflora* seedlings.

Dependent Variable	Independent Variable	*df*	*F*-Values	*p*-Values
Dry weight (g·plant^−1^)	A	3	27.83	<0.001
	AMF	1	4.72	<0.001
	A×AMF	3	0.47	0.064

**Table 2:** Relative concentration and fold changes of metabolites in *P. tenuiflora* seedlings under alkalinity stress after mycorrhizal inoculation.

Category of Metabolites	Kegg ID	Metabolite	Ionization Mode	Molecular Formula	*p*-Value	VIP ^a^	Fold Change ^b^
Amino acids and Amines	C00064	l-Glutamine	Q-TOF (+)/(−)	C_5_H_10_N_2_O_3_	0.00	1.71	2.42 ↑
	C00148	l-Proline	Q-TOF (−)	C_5_H_9_NO_2_	0.00	1.68	2.28 ↑
	C00152	l-Asparagine	Q-TOF (+)/(−)	C_4_H_8_N2O_3_	0.00	2.05	3.26 ↑
	C00188	l-Threonine	Q-TOF (+)	C_4_H_9_NO_3_	0.00	1.73	2.05 ↑
	C00263	l-homoserine	Q-TOF (−)	C_4_H_9_NO_3_	0.00	1.58	2.01 ↑
	C00449	l-Saccharopine	Q-TOF (+)/(−)	C_11_H_20_N_2_O_6_	0.00	1.73	2.93 ↑
	C01152	3-Methylhistidine	Q-TOF (+)	C_7_H_11_N_3_O_2_	0.00	2.65	6.78 ↑
	C01879	l-Pyroglutamic acid	Q-TOF (+)/(−)	C_5_H_7_NO_3_	0.00	1.59	2.34 ↑
	C05282	gamma-l-Glutamyl-l-glutamic acid	Q-TOF (+)	C_10_H_16_N_2_O_7_	0.00	1.49	2.06 ↑
	C06772	Diethanolamine	Q-TOF (+)	C_4_H_11_NO_2_	0.00	1.98	2.47 ↑
Carbohydrate and polyols	C00159	d-Mannose	Q-TOF (+)	C_6_H_12_O_6_	0.00	2.14	3.22 ↑
	C00474	Ribitol	Q-TOF (−)	C_5_H_12_O_5_	0.00	1.53	2.08 ↓
	C00476	d-Lyxose	Q-TOF (−)	C_5_H_10_O_5_	0.00	2.02	3.62 ↑
	C00860	l-Histidinol	Q-TOF (+)	C_6_H_11_N_3_O	0.00	1.46	2.22 ↑
	C01019	l-Fucose	Q-TOF (+)	C_6_H_12_O_5_	0.02	1.11	2.06 ↑
	C01083	Trehalose	Q-TOF (+)	C_12_H_22_O_11_	0.00	1.40	2.17 ↓
	C01613	Stachyose	Q-TOF (+)/(−)	C_24_H_42_O_21_	0.00	1.50	3.13 ↓
	C05235	Hydroxyacetone	Q-TOF (−)	C_3_H_6_O_2_	0.00	2.38	3.70 ↓
Organic acids	C00296	Quinate	Q-TOF (−)	C_7_H_12_O_6_	0.00	1.85	3.77 ↑
	C00493	Shikimic acid	Q-TOF (−)	C_7_H_10_O_5_	0.00	1.43	2.06 ↑
	C00499	Allantoate/Allantoic acid	Q-TOF (−)	C_4_H_8_N_4_O4	0.00	2.25	4.07 ↑
	C00847	4-Pyridoxic acid	Q-TOF (+)	C_8_H_9_NO_4_	0.01	1.30	2.22 ↓
	C00852	Chlorogenic acid	Q-TOF (−)	C_16_H_18_O_9_	0.00	2.31	4 ↓
	C01035	4-Guanidinobutyric acid	Q-TOF (+)	C_5_H_11_N_3_O_2_	0.00	2.40	3.55 ↑
	C01234	Cyclopropanecarboxylic acid	Q-TOF (+)	C_4_H_7_NO_2_	0.00	1.23	2.01 ↑
	C02835	Imidazoleacetic acid	Q-TOF (+)	C_5_H_6_N_2_O_2_	0.00	1.95	2.77 ↑
	C06104	Adipic acid	Q-TOF (+)	C_6_H_10_O_4_	0.00	1.72	2.13 ↓
	C12621	trans-3-Hydroxycinnamic acid	Q-TOF (+)	C_9_H_8_O_3_	0.00	1.77	2.14 ↑
	C00815	Citramalic acid	Q-TOF (+)	C_5_H_8_O_5_	0.00	1.31	2.63 ↑
Fatty acids	C00249	Palmitic acid	Q-TOF (−)	C_16_H_32_O_2_	0.00	2.28	4 ↓
	C08367	trans-Vaccenic acid	Q-TOF (+)	C_18_H_34_O_2_	0.00	1.66	2.38 ↓
Flavonoids	C09789	Naringin	Q-TOF (−)	C_27_H_32_O_14_	0.00	1.41	2.08 ↑
	C10084	Quercetin 3′-methyl ether	Q-TOF (+)	C_16_H_12_O_7_	0.00	3.07	16.67 ↓
	C10107	Myricetin	Q-TOF (+)	C_15_H_10_O_8_	0.00	1.59	2.04 ↓
	C10521	Prunetin	Q-TOF (+)	C_16_H_12_O_5_	0.00	1.88	3.04 ↑
	C12249	Astragalin	Q-TOF (+)	C_21_H_20_O_11_	0.00	1.64	2.56 ↓
	C12627	Apigenin 7-O-neohesperidoside	Q-TOF (+)	C_27_H_30_O_14_	0.00	2.74	9.26 ↑
	C14536	Glycitein	Q-TOF (+)/(−)	C_16_H_12_O_5_	0.00	1.70	2.53 ↑
	C00858	Formononetin	Q-TOF (+)	C_16_H_12_O_4_	0.00	1.87	2.64 ↑
Steroids	C01780	Aldosterone	Q-TOF (+)	C_21_H_28_O_5_	0.00	1.52	2.3 ↑
	C05122	Taurocholate	Q-TOF (−)	C_26_H_45_NO_7_S	0.00	1.75	2.47 ↑
Nucleic acids	C00147	Adenine	Q-TOF (−)	C_5_H_5_N_5_	0.00	1.77	2.63 ↓
	C00212	Adenosine	Q-TOF (+)	C_10_H_13_N_5_O_4_	0.00	1.63	2.13 ↑
	C00387	Guanosine	Q-TOF (+)/(−)	C_10_H_13_N_5_O_5_	0.00	2.03	2.73 ↑
	C00526	2′-Deoxyuridine	Q-TOF (−)	C_9_H_12_N_2_O_5_	0.00	2.13	3.66 ↑
	C00455	Nicotinamide ribotide	Q-TOF (−)	C_11_H_15_N_2_O_8_P	0.00	1.88	2.64 ↑
Phytohormones	C00805	Salicylic acid	Q-TOF (+)	C_7_H_6_O_3_	0.00	1.90	2.79 ↑
	C06082	(+)-Abscisic acid	Q-TOF (−)	C_15_H_20_O_4_	0.00	1.29	2.07 ↑
Others	C00258	Glyceric acid	Q-TOF (−)	C_3_H_6_O_4_	0.00	1.59	2.22 ↓
	C00279	D-Erythrose 4-phosphate	Q-TOF (+)	C_4_H_9_O_7_P	0.00	1.33	2.08 ↓
	C00670	Glycerophosphocholine	Q-TOF (+)	C_8_H_21_NO_6_P	0.00	1.83	2.3 ↑
	C00741	Diacetyl	Q-TOF (+)	C_4_H_6_O_2_	0.00	1.36	2.44 ↑
	C00755	Vanillin	Q-TOF (−)	C_8_H_8_O_3_	0.00	1.83	2.57 ↑
	C01551	Allantoin	Q-TOF (−)	C_4_H_6_N_4_O_3_	0.00	2.68	8.15 ↑
	C03068	3-Hydroxyhexobarbital	Q-TOF (+)	C_12_H_16_N_2_O_4_	0.00	1.64	2.32 ↓
	C03710	N-Benzyloxycarbonylglycine	Q-TOF (+)	C_10_H_11_NO_4_	0.00	1.60	2.74 ↑
	C04051	5-Amino-4-carbamoylimidazole	Q-TOF (−)	C4H6N4O	0.00	1.60	2.24 ↑
	C07042	Hydromorphone	Q-TOF (+)	C_17_H_19_NO_3_	0.00	2.97	14.28 ↓
	C07414	Pargyline	Q-TOF (+)	C_11_H_13_N	0.00	1.63	2.86 ↑
	C07537	Ethylmorphine	Q-TOF (+)	C_19_H_23_NO_3_	0.00	3.06	14.29 ↓
	C08313	3-Methylindole	Q-TOF (+)	C_9_H_9_N	0.00	1.78	2.09 ↑
	C11263	6-Benzylaminopurine	Q-TOF (+)	C_12_H_11_N_5_	0.00	2.61	5.64 ↑

^a^ VIP, variable importance in relation to project. ^b^ Relative metabolite level of non-AM *Puccinellia tenuiflora* leaves treated with NaHCO_3_ in comparison with the control (↑, increase, ↓, decrease).

## Supplementary Materials

The following are available online at https://www.mdpi.com/2076-2607/8/3/327/s1. Figure S1: Model overview (OPLS-DA) of metabolic profiles in non-AM seedlings in response to alkali stress (A,B), AM seedlings in response to alkalinity stress (C,D) and comparison in non-AM seedlings and AM seedlings under alkali stress (E,F) in positive (A,C,E) and negative modes (B,D,F). Figure S2: Heat map analysis combined with hierarchical cluster analysis of non-AM seedlings under control and non-AM seedlings under alkalinity stress. Figure S3: Heat map analysis combined with hierarchical cluster analysis of AM seedlings under control and non-AM seedlings under alkalinity stress. Figure S4: The metabolic model in *P. tenuiflora* seedlings after AMF inoculation under alkalinity stress (AM-AS vs. AS analysis) was visualized using the iPath v3.0 webserver, which maps the reactions of the metabolic model to KEGG metabolic pathways. It therefore does not capture metabolism not present in the KEGG pathways. The remarkable changed metabolic pathways were represented as a red line and the unchanged metabolic pathways were represented as a gray line. Red circles represented the differential metabolite, which direct mapping via selection of complete pathways, complete modules or reaction compound querying. Figure S5: The metabolic model in non-AM P. tenuiflora seedlings under alkali stress (AS vs. CK analysis) was visualized using the iPath v3.0 webserver, which maps the reactions of the metabolic model to KEGG metabolic pathways. It therefore does not capture metabolism not present in the KEGG pathways. The remarkable changed metabolic pathways were represented as a red line and the unchanged metabolic pathways were represented as a gray line. Red circles represented the differential metabolite, which direct mapping via selection of complete pathways, complete modules or reaction compound querying. Figure S6: The metabolic model in P. tenuiflora seedlings after AMF inoculation under alkalinity stress (AM-AS vs. AS analysis) was visualized using the iPath v3.0 webserver, which maps the reactions of the metabolic model to KEGG metabolic pathways. It therefore does not capture metabolism not present in the KEGG pathways. The remarkable changed metabolic pathways were represented as a red line and the unchanged metabolic pathways were represented as a gray line. Red circles represented the differential metabolite, which direct mapping via selection of complete pathways, complete modules or reaction compound querying. Table S1: Relative concentration and ratio changes of metabolites in the non-AM *Puccinellia tenuiflora* seedlings under alkali stress. Table S2: Relative concentration and ratio changes of metabolites in the AM *Puccinellia tenuiflora* seedlings under alkali stress.

## References

[1] Slama I., Abdelly C., Bouchereau A., Flowers T., Savoure A. (2015). Diversity, distribution and roles of osmoprotective compounds accumulated in halophytes under abiotic stress. Ann. Bot..

[2] Li M.N., Zhang K., Sun Y., Cui H.T., Cao S.H., Yan L. (2018). Growth, physiology, and transcriptional analysis of two contrasting *Carex rigescens* genotypes under salt stress reveals salt-tolerance mechanisms. J. Plant Physiol..

[3] Shelden M.C., Dias D.A., Jayasinghe N.S., Bacic A., Roessner U. (2016). Root spatial metabolite profiling of two genotypes of barley (*Hordeum Vulgare* L.) Reveals differences in response to short-term salt stress. J. Exp. Bot..

[4] Lin J.X., Wang Y.N., Sun S.N., Mu C.S., Yan X.F. (2017). Effects of arbuscular mycorrhizal fungi on the growth, photosynthesis and photosynthetic pigments of *Leymus Chinensis* seedlings under salt-alkali stress and nitrogen deposition. Sci. Total Environ..

[5] Yang C.W., Jianaer A., Li C.Y., Shi D.C., Wang D.L. (2008). Comparison of the effects of salt-stress and alkali-stress on photosynthesis and energy storage of an alkali-resistant halophyte *Chloris Virgata*. Photosynthetica.

[6] Yang C.W., Gao W.Q., Shi D.C. (2010). Physiological roles of organic acids in alkali-tolerance of the alkali-tolerant halophyte. Agron. J..

[7] Lin J.X., Yu D.F., Shi Y.J., Sheng H.C., Li C., Wang Y. (2016). Salt-alkali tolerance during germination and establishment of *Leymus Chinensis* in the songnen grassland of China. Ecol. Eng..

[8] Yang J.Y., Zheng W., Tian Y., Wu Y., Zhou D.W. (2011). Effects of various mixed salt-alkaline stresses on growth, photosynthesis, and photosynthetic pigment concentrations of *Medicago ruthenica* seedlings. Photosynthetica.

[9] Lin J.X., Peng X.Y., Hua X.Y., Sun S.N., Wang Y.N., Yan X.F. (2018). Effects of arbuscular mycorrhizal fungi on *Leymus Chinensis* seedlings under salt–alkali stress and nitrogen deposition conditions: From osmotic adjustment and ion balance. RSC Adv..

[10] Meng X., Zhao Q., Jin Y., Yu J., Yin Z., Chen S., Dai S.J. (2016). Chilling-responsive mechanisms in halophyte *Puccinellia Tenuiflora* seedlings revealed from proteomics analysis. J. Proteom..

[11] Yan X.F., Sun G.F. (2000). Physiological Ecology Research of Puccinellia tenuiflora.

[12] Wang Y., Sun G., Suo B., Chen G., Wang J., Yan Y. (2008). Effects of Na_2_CO_3_and NaCl stresses on the antioxidant enzymes of chloroplasts and chlorophyll fluorescence parameters of leaves of *Puccinellia Tenuiflora* (*Turcz*.) scribn.et merr. Acta Physiol. Plant..

[13] Guo L.Q., Shi D.C., Wang D.L. (2010). The key physiological response to alkali stress by the alkali-resistant halophyte *Puccinellia Tenuiflora* is the accumulation of large quantities of organic acids and into the rhyzosphere. J. Agron. Crop Sci..

[14] Liu H., Zhang X., Takano T., Liu S. (2009). Characterization of a *PutCAX*1 gene from *Puccinellia Tenuiflora* that confers Ca^2+^ and Ba^2+^ tolerance in yeast. Biochem. Biophys. Res. Commun..

[15] Ardie S.W., Liu S., Takano T. (2010). Expression of the AKT1-type K^+^ channel gene from *Puccinellia Tenuiflora*, *Put*AKT1, enhances salt tolerance inarabidopsis. Plant Cell Rep..

[16] Wang X., Geng S., Ri Y.J., Cao D., Yang C. (2011). Physiological responses and adaptive strategies of tomato plants to salt and alkali stresses. Sci. Hortic..

[17] Yu J., Chen S., Wang T., Sun G., Dai S. (2013). Comparative proteomic analysis of *Puccinellia tenuiflora* leaves under Na_2_CO_3_ stress. Int. J. Mol. Sci..

[18] Wu Q.S., Zou Y.N., He X.H. (2013). Mycorrhizal symbiosis enhances tolerance to NaCl stress through selective absorption but not selective transport of K^+^ over Na^+^ in trifoliate orange. Sci. Hortic..

[19] Wu Q.S., Zou Y.N., He X.H. (2010). Contributions of arbuscular mycorrhizal fungi to growth, photosynthesis, root morphology and ionic balance of citrus seedlings under salt stress. Acta Physiol. Plant..

[20] Rillig M.C., Mummey D.L. (2006). Mycorrhizas and soil structure. New Phytologist..

[21] Asrar A.W.A., Elhindi K.M. (2011). Alleviation of drought stress of marigold (*Tagetes erecta*) plants by using arbuscular mycorrhizal fungi. Saudi J. Biol. Sci..

[22] Porcel R., Aroca R., Ruiz-Lozano J.M. (2012). Salinity stress alleviation using arbuscular mycorrhizal fungi: A review. Agron. Sustain. Dev..

[23] Liu Z.L., Li Y.J., Hou H.Y., Zhu X.C., Rai V., He X.Y., Tian C.J. (2013). Differences in the arbuscular mycorrhizal fungi-improved rice resistance to low temperature at two N levels: Aspects of N and C metabolism on the plant side. Plant Physiol. Biochem..

[24] Kumar A., Sharma S., Mishra S., Dames J.F. (2015). Arbuscular mycorrhizal inoculation improves growth and antioxidative response of *Latropha Curcas* (L.) under Na_2_SO_4_ salt stress. G. Bot. Ital..

[25] Ouziad F., Wilde P., Schmelzer E., Hildebrandt U., Bothe H. (2006). Analysis of expression of aquaporins and Na^+^/H^+^ transporters in tomato colonized by arbuscular mycorrhizal fungi and affected by salt stress. Environ. Exp. Bot..

[26] He Z.Q., He C.X., Yan Y., Zhang Z.B., Wang H.S., Li H.X., Tang H.R. (2011). Regulative effect of arbuscular mycorrhizal fungi on water absorption and expression of aquaporin genes in tomato under salt stress. Acta Hortic. Sin..

[27] Jie C., Haoqiang Z., Xinlu Z., Ming T. (2017). Arbuscular mycorrhizal symbiosis alleviates salt stress in black locust through improved photosynthesis, water status, and K^+^/Na^+^ homeostasis. Front. Plant Sci..

[28] Farag M.A., Andrea P., Wessjohann L.A. (2009). Comparative metabolite profiling and fingerprinting of medicinal licorice roots using a multiplex approach of GC-MS, LC-MS and 1D NMR techniques. Phytochemistry.

[29] Mamdouh M., Khedr A.H.A., Serag M.M., Abu-Alnaga A.Z., Nada R.M. (2012). Regulation of metabolomics in *Atriplex halimus* growth under salt and drought stress. Plant Growth Regul..

[30] Yang D.S., Zhang J., Li M.X., Shi L.X. (2017). Metabolomics analysis reveals the salt-tolerant mechanism in *Glycine soja*. J. Plant Growth Regul..

[31] Lyu X., Ng K.R., Mark R., Lee J.L., Chen W. (2018). Comparative metabolic profiling of engineered Saccharomyces cerevisiae with enhanced flavonoids production. J. Funct. Foods..

[32] Wu D., Cai S., Chen M., Ye L., Chen Z., Zhang H. (2013). Tissue metabolic responses to salt stress in wild and cultivated barley. PLoS ONE.

[33] Nam M., Bang E., Taek K., Yuran K., Eun K., Kyungwon C. (2015). Metabolite profiling of diverse rice germplasm and identification of conserved metabolic markers of rice roots, in response to long-term mild salinity stress. Int. J. Mol. Sci..

[34] Pang Q.Y., Zhang A.Q., Zang W., Wei L., Yan X.F. (2016). Integrated proteomics and metabolomics for dissecting the mechanism of global responses to salt and alkali stress in *Suaeda Corniculata*. Plant Soil..

[35] Guo R., Shi L.X., Yan C., Zhong X., Gu F.X., Liu Q., Xia X., Li H. (2017). Ionomic and metabolic responses to neutral salt or alkaline salt stresses in maize (*Zea mays* L.) seedlings. BMC Plant Biol..

[36] Phillips J., Hayman D. (1970). Improved procedures for clearing roots and staining parasitic and vesicular-arbuscular mycorrhizal fungi for rapid assessment of infection. Trans. Br. Mycol. Soc..

[37] Wu N., Li Z., Liu H., Tang M. (2015). Influence of arbuscular mycorrhiza on photosynthesis and water status of *Populus cathayana* Rehder males and females under salt stress. Acta Physiol. Plant..

[38] Zhang C., Wang W., Lu R., Jin S., Chen Y., Fan M. (2016). Metabolic responses of *Beauveria Bassiana* to hydrogen peroxide-induced oxidative stress using an LC-MS-based metabolomics approach. J. Invertebr. Pathol..

[39] Hajiboland R., Aliasgharzadeh N., Laiegh S.F., Poschenrieder C. (2010). Colonization with arbuscular mycorrhizal fungi improves salinity tolerance of tomato (*Solanum Lycopersicum* L.) plants. Plant Soil.

[40] Wang Y.N., Jie W.G., Peng X.Y., Hua X.Y., Yan X.F., Zhou Z.Q., Lin J.X. (2019). Physiological adaptive strategies of oil seed crop *Ricinus communis* early seedlings (cotyledon vs. true leaf) under salt and alkali stresses: From the growth, photosynthesis and chlorophyll fluorescence. Front. Plant Sci..

[41] Feng G., Zhang F., Li X., Tian C., Tang C., Rengel Z. (2002). Improved tolerance of maize plants to salt stress by arbuscular mycorrhiza is related to higher accumulation of soluble sugars in roots. Mycorrhiza.

[42] Jahromi F., Aroca R., Porcel R., Ruiz-Lozano J.M. (2008). Influence of salinity on the *In Vitro* development of *Glomus Intraradices* and on the *In Vitro* physiological and molecular responses of mycorrhizal lettuce plants. Microb. Ecol..

[43] Eftekhari M., Alizadeh M., Ebrahimi P. (2012). Evaluation of the total phenolics and quercetin content of foliage in mycorrhizal grape (*Vitis Vinifera* L.) varieties and effect of postharvest drying on quercetin yield. Ind. Crops Prod..

[44] Wu Q.S., Zou Y.N., Huang Y.M., Li Y., He X.H. (2013). Arbuscular mycorrhizal fungi induce sucrose cleavage for carbon supply of arbuscular mycorrhizas in citrus genotypes. Sci. Hortic..

[45] Less H., Galili G. (2008). Principal transcriptional programs regulating plant amino acid metabolism in response to abiotic stresses. Plant Physiol..

[46] Xiao Q., Zheng H.L., Chen Y., Huang W.B., Zhu Z. (2005). Effects of salinity on the growth and proline, soluble sugar and protein contents of *Spartina alterniflora*. Chin. J. Ecol..

[47] Tian X.Y., Liu Y.J., Guo Y.C. (2008). Effect of salt stress on Na^+^,K^+^, proline, soluble sugar and protein of NHC. Pratacult. Sci..

[48] Yang C., Shi D., Wang D. (2008). Comparative effects of salt and alkali stresses on growth, osmotic adjustment and ionic balance of an alkali-resistant halophyte *Suaeda Glauca* (Bge.). Plant Growth Regul..

[49] Lin J.X., Li Z., Mu C., Wang Y., Li X. (2014). Physiological adaptive mechanisms of *Leymus Chinensis* during germination and early seedling stages under saline and alkaline conditions. J. Anim. Plant Sci..

[50] Li M.X., Guo R., Yang J., Jin X.F., Zhang H.Y., Shi L.X. (2017). Comparison of salt tolerance in *soja* based on metabolomics of seedling roots. Front. Plant Sci..

[51] Alqarawi A.A., Hashem A., Abd Allah E.F., Alshahrani T.S., Huqail A.A. (2014). Effect of salinity on moisture content, pigment system, and lipid composition in *Ephedra alata* Decne. Acta Biol. Hung..

[52] Song T.T., X H.H., Na S., Liu J., Pu T., Yang Y.Y. (2017). Metabolomic analysis of alfalfa (*Medicago Sativa* L.) root-symbiotic rhizobia responses under alkali stress. Front. Plant Sci..

[53] Widodo J., Patterson J.H., Newbigin E., Tester M., Bacic A., Roessner U. (2009). Metabolic responses to salt stress of barley (*Hordeum vulgare* L.) cultivars, sahara and clipper, which differ in salinity tolerance. J. Exp. Bot..

[54] Tavakoli M., Poustini K., Alizadeh H. (2018). Proline accumulation and related genes in wheat leaves under salinity stress. J. Agric. Sci. Technol..

[55] Jiao Y., Bai Z., Xu J., Zhao M., Khan Y., Hu Y., Shi L. (2018). Metabolomics and its physiological regulation process reveal the salt-tolerant mechanism in *Glycine Soja* seedling roots. Plant Physiol. Biochem..

[56] Boriboonkaset T., Theerawitaya C., Yamada N., Pichakum A., Supaibulwatana K., Cha-Um S. (2013). Regulation of some carbohydrate metabolism-related genes, starch and soluble sugar contents, photosynthetic activities and yield attributes of two contrasting rice genotypes subjected to salt stress. Protoplasma.

[57] Kerepesi I., Galiba G. (2000). Osmotic and salt stress-induced alteration in soluble carbohydrate content in wheat seedlings. Crop Sci..

[58] Janz D., Behnke K., Schnitzler J.P., Kanawati B., Schmitt-Kopplin P., Polle A. (2010). Pathway analysis of the transcriptome and metabolome of salt sensitive and tolerant poplar species reveals evolutionary adaption of stress tolerance mechanisms. BMC Plant Biol..

[59] Zhang J., Yang D., Li M., Shi L. (2016). Metabolic profiles reveal changes in wild and cultivated soybean seedling leaves under salt stress. PLoS ONE.

[60] McNeil S.D., Nuccio M.L., Hanson A.D. (1999). Betaines and related osmoprotectants: Targets for metabolic engineering of stress resistance. Plant Physiol..

[61] Rui G., Lianxuan S., Chunwu Y., Changrong Y., Xiuli Z., Qi L. (2016). Comparison of ionomic and metabolites response under alkali stress in old and young leaves of cotton (*Gossypium Hirsutum* L.) seedlings. Front. Plant Sci..

[62] Shunsuke W., Mayumi M., Yuki H., Hiroshi T., Hiroshi S., Atsushi S. (2014). The purine metabolite allantoin enhances abiotic stress tolerance through synergistic activation of abscisic acid metabolism. Plant Cell Environ..

[63] Werner A.K., Witte C.P. (2011). The biochemistry of nitrogen mobilization: Purine ring catabolism. Trends Plant Sci..

[64] Yobi A., Wone B.W., Xu W., Alexander D.C., Guo L., Ryals J.A., Melvin J.O., Cushman J.C. (2013). Metabolomic profiling in *Selaginella lepidophylla* at various hydration states provides new insights into the mechanistic basis of desiccation tolerance. Mol. Plant.

[65] Wang F., Kong W., Wong G., Fu L., Peng R., Li Z., Yao Q. (2016). *ATMYB12* regulates flavonoids accumulation and abiotic stress tolerance in transgenic *Arabidopsis Thaliana*. Mol. Genet. Genom..

[66] YGao J.J., Zhang Z., Peng R.H., Xiong A.S., Xu J., Zhu B. (2011). Forced expression of *Mdmyb10*, a myb transcription factor gene from apple, enhances tolerance to osmotic stress in transgenic arabidopsis. Mol. Biol. Rep..

[67] Kumar M.S.S., Mawlong I., Ali K., Tyagi A. (2018). Regulation of phytosterol biosynthetic pathway during drought stress in rice. Plant Physiol. Biochem..

[68] Lee S.J., Jeong E.M., Ki A.Y., Oh K.S., Kwon J., Jeong J.H. (2016). Oxidative defense metabolites induced by salinity stress in roots of *Salicornia Herbacea*. J. Plant Physiol..

[69] Ryu H., Cho Y.G. (2015). Plant hormones in salt stress tolerance. J. Plant Biol..

[70] Gunes A., Inal A., Alpaslan M., Cicek N., Guneri E., Eraslan F. (2005). Effects of exogenously applied salicylic acid on the induction of multiple stress tolerance and mineral nutrition in maize (*Zea Mays* L.). Arch. Agron. Soil Sci..

[71] Stevens J., Senaratna T., Sivasithamparam K. (2006). Salicylic acid induces salinity tolerance in tomato (*Lycopersicon esculentum* cv. Roma): Associated changes in gas exchange, water relations and membrane stabilisation. Plant Growth Regul..

[72] Hashem A., Alqarawi A.A., Radhakrishnan R., Al-Arjani A.B.F., Aldehaish H.A., Egamberdieva D. (2018). Arbuscular mycorrhizal fungi regulate the oxidative system, hormones and ionic equilibrium to trigger salt stress tolerance in, *Cucumis sativus* L.. Saudi J. Biol. Sci..

[73] Liu H.G., Wang Y.J., Hart M., Chen H., Tang M. (2016). Arbuscular mycorrhizal symbiosis regulates hormone and osmotic equilibrium of *Lycium barbarum* L. under salt stress. Mycosphere.

