# Supplementary material for: Metabolomics Analysis Reveals the Alkali Tolerance Mechanism in Puccinellia tenuiflora Plants Inoculated with Arbuscular Mycorrhizal Fungi
Source: Microorganisms. 2020 Feb 26;8(3):327. doi: 10.3390/microorganisms8030327 (PMC7142761; doi:10.3390/microorganisms8030327)
Supplement: Supplementary file 1 [file microorganisms-08-00327-s001.zip › supplementary/Fig S.doc]

**Fig. S1 Model overview (OPLS-DA) of metabolic profiles in non-AM seedlings in response to alkali stress (A, B), AM seedlings in response to alkalinity stress (C, D) and comparison in non-AM seedlings and AM seedlings under alkali stress (E, F) in positive (A, C, E) and negative modes (B, D, F).**

**Fig. S2 Heat map analysis combined with hierarchical cluster analysis of non-AM seedlings under control and non-AM seedlings under alkalinity stress.**

**Fig. S3 Heat map analysis combined with hierarchical cluster analysis of AM seedlings under control and non-AM seedlings under alkalinity stress.**

**Fig. S4 The metabolic model in *P. tenuiflora* seedlings after AMF inoculation under alkalinity stress (AM-AS vs. AS [analysis](../../../../C:/Users/Administrator/AppData/Local/youdao/dict/Application/8.5.1.0/resultui/html/index.html#/javascript:;)) was visualized using the iPath v3.0 webserver, which maps the reactions of the metabolic model to KEGG metabolic pathways. It therefore does not capture metabolism not present in the KEGG pathways. The remarkable changed metabolic pathways were represented as a red line and the unchanged metabolic pathways were represented as a gray line. Red circles represented the differential metabolite, which direct mapping via selection of complete pathways, complete modules or reaction compound querying. C00064: L-Glutamine; C00147: Adenine; C00148: L-Proline; C00152: L-Asparagine; C00159: D-Mannose; C00188: L-Threonine; C00212: Adenosine; C00249: Palmitic acid; C00258: Glyceric acid; C00263: L-homoserine C00279: D-Erythrose 4-phosphate; C00387: Guanosine C00449: L-Saccharopine; C00455: Nicotinamide ribotide; C00474: Ribitol; C00493: Shikimic acid; C00499: Allantoate/Allantoic acid; C00755: Vanillin; C00805: Salicylic acid; C00847: 4-Pyridoxic acid; C00860: L-Histidinol C01019: L-Fucose; C01083: Trehalose; C01234: Cyclopropanecarboxylic acid; C01780: Aldosterone; C06082: (+)-Abscisic acid; C06104: Adipic acid;**

**Fig. S5 The metabolic model in non-AM *P. tenuiflora* seedlings under alkali stress (AS vs. CK [analysis](../../../../C:/Users/Administrator/AppData/Local/youdao/dict/Application/8.5.1.0/resultui/html/index.html#/javascript:;)) was visualized using the iPath v3.0 webserver, which maps the reactions of the metabolic model to KEGG metabolic pathways. It therefore does not capture metabolism not present in the KEGG pathways. The remarkable changed metabolic pathways were represented as a red line and the unchanged metabolic pathways were represented as a gray line. Red circles represented the differential metabolite, which direct mapping via selection of complete pathways, complete modules or reaction compound querying. C00049: L-Aspartate; C00062: L-Arginine; C00089: Sucrose; C00122: Fumaric acid; C00135: L-Histidine; C00147: Adenine; C00149: Malic acid; C00152: L-Asparagine; C00156: 4-Hydroxybenzoate; C00158: Citrate; C00188: L-Threonine; C00208: D-Maltose; C00212: Adenosine; C00219: Arachidonic acid; C00243: D-Lactose; C00259: L-Arabinose; C00280: Androstendione; C00314: Pyridoxine; C00330: Deoxyguanosine; C00333: D-galacturonic acid; C00346: O-Phosphoethanolamine; C00378: Thiamine; C00387: Guanosine; C00407: L-Isoleucine; C00455: Nicotinamide ribotide; C00499: Allantoate; C00755: Vanillin; C01780: Aldosterone; C00805: Salicylic acid; C00860: L-Histidinol; C00882: Dephosphocoenzyme A; C00954: Indoleacetic acid; C00956: DL-2-Aminoadipic acid; C01035: 4-Guanidinobutyric acid; C01083: Trehalose; C01234: Cyclopropanecarboxylic acid; C01780:Aldosterone; C02291: L-Cystathionine; C05122: Taurocholate; C05122: Taurocholate; C05488: 11-Deoxycortisol; C05625: Rutin; C05635: 5-Hydroxyindoleacetate; C06082: (+)-Abscisic acid; C06427: alpha-Linolenic acid;**

**Fig. S6 The metabolic model in *P. tenuiflora* seedlings after AMF inoculation under alkalinity stress (AM-AS vs. AS [analysis](../../../../C:/Users/Administrator/AppData/Local/youdao/dict/Application/8.5.1.0/resultui/html/index.html#/javascript:;)) was visualized using the iPath v3.0 webserver, which maps the reactions of the metabolic model to KEGG metabolic pathways. It therefore does not capture metabolism not present in the KEGG pathways. The remarkable changed metabolic pathways were represented as a red line and the unchanged metabolic pathways were represented as a gray line. Red circles represented the differential metabolite, which direct mapping via selection of complete pathways, complete modules or reaction compound querying. C00064: L-Glutamine; C00147: Adenine; C00148: L-Proline; C00152: L-Asparagine; C00159: D-Mannose; C00188: L-Threonine; C00212: Adenosine; C00249: Palmitic acid; C00258: Glyceric acid; C00263: L-homoserine C00279: D-Erythrose 4-phosphate; C00387: Guanosine C00449: L-Saccharopine; C00455: Nicotinamide ribotide; C00474: Ribitol; C00493: Shikimic acid; C00499: Allantoate/Allantoic acid; C00755: Vanillin; C00805: Salicylic acid; C00847: 4-Pyridoxic acid; C00860: L-Histidinol C01019: L-Fucose; C01083: Trehalose; C01234: Cyclopropanecarboxylic acid; C01780: Aldosterone; C06082: (+)-Abscisic acid; C06104: Adipic acid;**
